# Pandemic and vaccine coverage: challenges of returning to schools

**DOI:** 10.11606/s1518-8787.2020054003142

**Published:** 2020-11-05

**Authors:** Ana Paula Sayuri Sato

**Affiliations:** 1 Universidade de São Paulo Faculdade de Saúde Pública Departamento de Epidemiologia São PauloSP Brasil Universidade de São Paulo. Faculdade de Saúde Pública. Departamento de Epidemiologia. São Paulo, SP, Brasil

**Keywords:** Coronavirus Infections, prevention & control, Vaccine-Preventable Diseases, Immunization Coverage, Immunization Programs

## Abstract

Since March 2020, Brazil has faced the pandemic of the coronavirus disease 2019 (Covid-19), which has severely modified the way in which the population lives and uses health services. As such, face-to-face attendance has dropped dramatically, even for child vaccination, due to measures of social distancing to mitigate the transmission of the virus. Several countries have recorded a substantial drop in vaccination coverage in children, especially of those under two years of age. In Brazil, administrative data indicate the impact of the covid-19 pandemic on this downward trend, which was already an important challenge of the National Immunization Program in recent years. Many children will be susceptible to immunopreventable diseases, which reinforces the need to assess the vaccine status of schoolchildren before returning to face-to-face classes.

## INTRODUCTION

Vaccination (along with other public policies, especially those aimed at expanding sanitation) has made possible to substantially decrease the number of deaths of children under five years of age worldwide^1^. Widespread vaccination allowed the eradication or control of immunopreventable diseases in several regions of the world, including Brazil, due to successful immunization programs^1^^–^^3^.

In Brazil, since the 1990s, vaccine coverage in children under one year of age had rates above 95%, which indicated the high participation of the population in vaccination and the good performance of the National Immunization Program (NIP)^4^. The gradual implementation of the Brazilian Unified Health System (SUS) in the late 1980s allowed for a high rate of vaccine coverage through the expansion and decentralization of health services, mainly due to its principle of universal and free access to vaccination^5^^–^^6^.

Throughout its history, the NIP faced several challenges. In the 1980s, the first national surveys of vaccination coverage showed worse coverage in poorer segments of the population; this difference disappeared in the late 1990s, indicating that equity of access to vaccination had been reached in different socioeconomic strata of Brazil^2^^,^^7^^,^^8^. However, according to the 2007 national survey, the country now has a lower coverage on both the richer and the extremely poor demographics^9^. Moreover, from 2016 onwards vaccine coverage rates have declined about 10% to 20%^10^^,^^11^, due to factors not yet understood. The measles epidemic that hit several states in 2018 and 2019 is an immediate consequence of the decrease in vaccine coverage^12^.

Among the possible explanations for this, we have the decrease in the perception of risk of these diseases and the increased perception of risk of adverse events following immunization (AEFI). This phenomenon was also recorded in other countries, due to the success of immunization programs when disease control or elimination is reached, a result of the prolonged maintenance of high vaccination coverage. Thus, success itself has become a great challenge^13^.

However, it is accepted that this is not the sole reason: among other factors that influenced the drop in vaccination coverage since 2016, the emergence of vaccine hesitancy is highlighted. This a phenomenon that has gained importance in various parts of the world and is characterized by the delay in accepting or refusal of the vaccine, regardless of its availability and access to health services^10^^,^^14^^–^^16^.

The political and economic crisis, the decrease in government support for the SUS^17^ and the dissemination by social networks of distorted information about vaccines are also worthy of mention, all of which possibly contributed to the sharp drop in vaccine coverage in recent years^18^^–^^20^.

## THE GLOBAL IMPACT OF PANDEMIC ON CHILD VACCINATION

In 2020, due to the pandemic of coronavirus disease 2019 (covid-19), face-to-face attendance in health services dropped dramatically in many countries; this included child vaccination, given the measures of social distancing to mitigate viral transmission^21^^–^^27^.

Efforts to contain the pandemic, which involve distant medicine practices and the use of other technologies in order to continue health care at home, have affected vaccination actions, which require travels to the healthcare unit^22^. Parental concern in exposing children to Sars-CoV-2 when taking them to health services for vaccination also contributed to the decline in vaccination coverage^22^^,^^24^^–^^26^^,^^28^. A risk-benefit study in African countries showed that avoidable deaths from routine vaccination outweigh the excess risk of death from covid-19 associated with attendance at the healthcare unit, evidencing the need for increasing vaccination coverage at this time^21^.

Child vaccination coverage has declined sharply during the pandemic in several regions of the world^26^^,^^28^. In the USA, a considerable decline in the vaccine coverage of children was found, starting in the week after the national emergency scenario was declared (March 13, 2020). Higher rates were found among children under two years of age^25^. In England, three weeks after the introduction of social distancing (March 20, 2020), there was a 19.8% drop in doses of the measles-mumps-rubella vaccine, compared to the same period in 2019^24^. In Michigan (USA), completeness of the vaccination schedule for five-year olds dropped from 67.0% to 49.7% in May 2020. At 16 months, it was found that measles vaccine coverage decreased from 76.1% to 70.9%^22^. In Indonesia, where immunization occurs in schools, a significant drop in coverage of the basic vaccination schedule was predicted after the closure of schools in March 2020^27^. Moreover, it is known that this impact will be even more important in families with unfavorable socioeconomic conditions^26^.

The World Health Organization (WHO) estimates that at least 80 million children will be susceptible to immunopreventable diseases such as measles, diphtheria and polio because of the decrease in vaccination coverage during the covid-19 pandemic^29^. It is worth remembering that outbreaks of measles were attributed to the interruption of vaccination services during the 2013–2016 Ebola epidemic in West Africa, causing a second public health crisis^30^^–^^31^.

The pandemic of the new coronavirus has challenged health systems around the world in providing essential services, including immunization programs, as routine vaccination and mass vaccination campaigns could contribute to the spread of covid-19^32^.

On March 26, 2020, WHO and the Pan American Health Organization published recommendations on vaccination during the covid-19 pandemic. The measures considered three scenarios of availability of health services and included the temporary suspension of mass vaccination campaigns during this period. It was recommended that routine vaccination be maintained in places where essential health services had operational capacity of human resources and supply of preserved vaccines, respecting social distancing and other measures to control transmission of Sars-CoV-2^33^^,^^34^.

In Brazil there was the recommendation of suspending routine immunization during the first 15 days of the influenza vaccination campaign, as this was a period in which older adults and health professionals were supposed to be vaccinated; although this was valid as a safety measure for the older population, it has generated concern among Brazilian medical societies^35^.

The WHO recognizes this fragility and recommends efforts to ensure high vaccination coverage, seeking herd immunization for preventable diseases, in such way that vaccination programs should adopt innovative measures^36^^,^^37^. Vaccination strategies in vehicles, at home or in specific rooms and well-separated from the locations of other clinical visits could be used, as well as the identification of absentees and recruitment for vaccination with the aid of electronic immunization registries (EIR)^22^^,^^24^^–^^26^^,^^28^.

EIR allow greater efficiency of health services, because, in addition to providing the evaluation of vaccination coverage, they also help in routine practice and enable the convocation of absentees, thus increasing the scope of immunization^38^^,^^39^. In addition, they are important sources of information, which can be applied in the evaluation of performance indicators and in the development of epidemiological research^38^^,^^40^. In 2020, researchers from countries such as the USA and the United Kingdom evaluated in real time the decline in vaccination coverage and the number of doses applied during the covid-19 pandemic through EIR. With this quick identification, it is possible to quickly adopt strategies in the face of this challenge^22^^,^^24^^,^^25^.

With the emergence of many radical groups worldwide that deny the importance of the pandemic and its associated mitigation measures, vaccine hesitancy might acquire more strength, especially considering the availability of vaccines for covid-19 in the near future that will have an important role in dealing with this disease^41^.

## CHALLENGES OF RETURNING TO SCHOOLS

The safe resumption of day care centers and schools should be a national priority. Children have lost fundamental benefits of social, educational and developmental nature. For many parents, it will not be possible to return to work if these institutions remain closed, thus exacerbating social inequities. Several individual practices (use of masks, hygiene, social distancing, temperature measurement etc) as well as environmental ones (maximum capacity and layout of classrooms, cleaning etc) will be necessary to prevent the transmission of Sars-CoV-2 between schoolchildren and staff, including in transportation to schools^42^^,^^43^.

However, in addition to care for covid-19^44^, the American Academy of Pediatrics recommended that schools, health services, and local health authorities promote child vaccination well before the beginning of the school year. It is important that children receive vaccines at the recommended age and be updated in case of vaccine delay due to the pandemic^45^. This recommendation should be considered in other countries, including Brazil.

In Brazil, the pandemic was an additional challenge for the return to schools due to the abovementioned immunopreventable diseases, as we recently faced a consistent drop in vaccination coverage and a wide epidemic of measles that reached several states and amounted for thousands of cases. This situation has worsened in 2020, which until August had registered more than 7,000 confirmed cases of measles^46^.

According to data from the NIP Information System (IS-NIP), when comparing the number of first doses of the pentavalent vaccine applied in March 2020 with March 2019, we found a decrease of 27% (Figure)^10^^–^^11^. These data indicate that the return to classes may increase the risk not only of the expansion of measles epidemics throughout the country, but also of the reemergence of other already controlled diseases, such as diphtheria and the whooping cough. Studies show that outbreaks of diphtheria occur when vaccination coverage drops due to migration and/or political instability, emphasizing that it is a disease of relevant lethality^47^^–^^49^.

**Figure:**
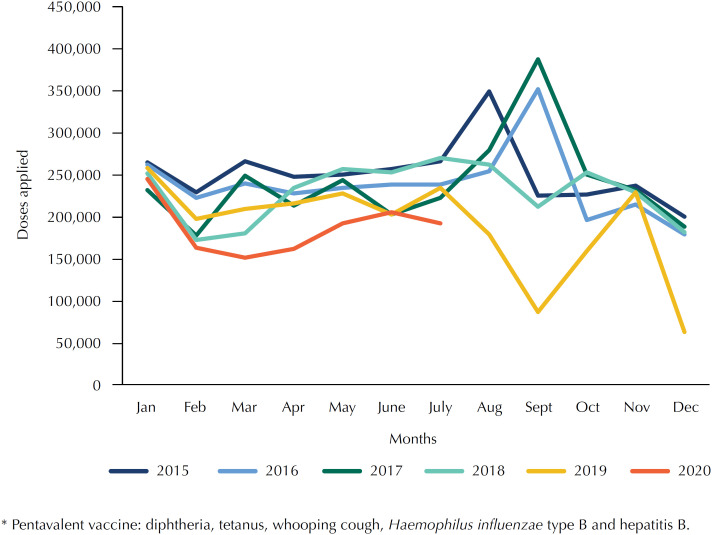
Number of doses applied of the pentavalent vaccine* (first dose) in Brazil, month and year (2015–2020).

Source: SI-PNI (http://sipni.datasus.gov.br).

Thus, it is evident that, before the progressive return of face-to-face school activities, intensive actions to assess the vaccine situation of this population will be necessary in order to recover sufficient vaccination coverage to prevent or reduce the spread of immunopreventable diseases^45^. Innovative instruments, such as EIR, can be useful for real-time assessment of vaccination coverage, as well as to warn about immunization and rescue individuals with vaccine delay^22^^,^^24^^,^^25^^,^^38^^–^^40^.

## FINAL REMARKS

To date, there are no studies on the impact of covid-19 on the decline in vaccine coverage. Delays in child vaccination (a demographic that should have been immunized in the most intense moment of social distancing) are also yet to be studied, even in other countries. Moreover, despite the universal access to child vaccination achieved by the NIP in the last decade, this impact will probably be greater in children from families with unfavorable socioeconomic conditions, due to less access to health services and information.

When social distancing measures are loosened, many children will be susceptible to preventable diseases, and there will be a need to assess the vaccine situation of schoolchildren before returning to school^22^^,^^24^^–^^26^^,^^28^^,^^45^.

The covid-19 pandemic recalled the importance of vaccination by showing how fast a disease can spread and cause irreparable harm in societies without this defense. When a safe and effective vaccine for Sars-CoV-2 is available, immunization programs will have an even greater challenge of strengthening and reaching those most vulnerable^28^.
