# Supplementary material for: Pandemic and vaccine coverage: challenges of returning to schools
Source: Rev Saude Publica. 2020 Nov 5;54:115. doi: 10.11606/s1518-8787.2020054003142 (PMC7647469; doi:10.11606/s1518-8787.2020054003142)
Supplement: Translation

# Translation: Pandemia e coberturas vacinais: desafios para o retorno às escolas

**Authors:** Ana Paula Sayuri Sato

**Affiliations:** 2 São PauloSP Brasil Universidade de São Paulo. Faculdade de Saúde Pública. Departamento de Epidemiologia. São Paulo, SP, Brasil

**Keywords:** Infecções por Coronavirus, prevenção & controle, Doenças Preveníveis por Vacina, Cobertura Vacinal, Programas de Imunização

## Abstract

Desde março de 2020, o Brasil enfrenta a pandemia da doença do coronavírus 2019 (covid-19), que modificou intensamente o modo de viver da população e o uso dos serviços de saúde, nos quais o comparecimento presencial caiu drasticamente, inclusive para a vacinação infantil, devido às medidas de distanciamento social para mitigar a transmissão do vírus. Diversos países registraram queda substancial das coberturas vacinais em crianças, especialmente nas menores de dois anos de idade. No Brasil, dados administrativos apontam o impacto da pandemia de covid-19 no agravamento dessa queda, que já constituía um desafio importante do Programa Nacional de Imunizações nos últimos anos. Muitas crianças estarão suscetíveis a doenças imunopreveníveis, o que reforça a necessidade de avaliar a situação vacinal dos escolares antes do retorno às aulas presenciais.

## INTRODUÇÃO

A vacinação, junto com outras políticas públicas, especialmente as voltadas à ampliação do saneamento, tornou possível o declínio substancial das mortes de crianças menores de cinco anos de idade em todo o globo^1^. O amplo uso da vacinação possibilitou erradicar, eliminar ou controlar doenças imunopreveníveis em diversas regiões do mundo, inclusive no Brasil, refletindo programas de imunização bem-sucedidos^1^^–^^3^.

No Brasil, desde a década de 1990, as coberturas vacinais em menores de um ano estiveram acima de 95%, o que indicava a elevada adesão da população brasileira à vacinação e o bom desempenho do Programa Nacional de Imunização (PNI)^4^. Vale destacar o papel da progressiva implantação do Sistema Único de Saúde (SUS), a partir do final da década de 1980, para o alcance das altas coberturas vacinais, por meio da expansão e descentralização dos serviços de saúde, mas principalmente pelo princípio do acesso universal e gratuito à vacinação^5^^–^^6^.

Em sua trajetória, o PNI enfrentou vários desafios. Nos anos 1980, os primeiros inquéritos nacionais de cobertura vacinal apontavam piores coberturas em segmentos mais pobres da população, diferença que desapareceu no final dos anos 1990, indicando que se havia atingido a equidade de acesso à vacinação em diferentes estratos socioeconômicos do Brasil^2^^,^^7^^,^^8^. No entanto, conforme o inquérito nacional de 2007, passamos a ter menores coberturas nos segmentos mais ricos e nos extremamente pobres da população^9^ e, a partir de 2016, por fatores ainda não perfeitamente compreendidos, as coberturas vacinais declinaram cerca de 10 a 20 pontos percentuais^10^^,^^11^. As epidemias de sarampo que atingiram vários estados em 2018 e 2019 são consequência imediata da diminuição das coberturas vacinais^12^.

Entre as possíveis explicações para isso, temos a diminuição da percepção de risco dessas doenças e o aumento da percepção de risco de eventos adversos pós-vacina (EAPV). Esse fenômeno também foi registrado em outros países, em decorrência do sucesso dos programas de imunização ao atingirem o controle ou a eliminação de doenças, como resultado da manutenção por tempo prolongado de elevadas coberturas vacinais. Ou seja, o sucesso passou a ser o seu grande desafio^13^.

No entanto, aceita-se que essa não seja a única razão: entre outros fatores que influenciaram a queda das coberturas vacinais a partir de 2016, é apontado o surgimento da hesitação vacinal, fenômeno que tem ganhado importância em várias partes do globo e se caracteriza pelo atraso em aceitar ou pela recusa da vacina, independentemente de sua disponibilidade e do acesso aos serviços de saúde^10^^,^^14^^–^^16^.

São citadas também a crise político-econômica, a diminuição do apoio governamental ao SUS^17^ e a difusão por redes sociais de informações distorcidas sobre vacinas, que possivelmente contribuíram para a acentuada queda das coberturas nos últimos anos^18^^–^^20^.

## O IMPACTO GLOBAL DA PANDEMIA NA VACINAÇÃO INFANTIL

Em 2020, com a pandemia da doença do coronavírus 2019 (covid-19), o comparecimento presencial nos serviços de saúde caiu drasticamente em muitos países, inclusive para a vacinação infantil, devido às medidas de distanciamento social para mitigar a transmissão do vírus^21^^–^^27^.

Os esforços para conter a pandemia, que envolvem práticas de telemedicina e o uso de outras tecnologias a fim de dar continuidade aos cuidados de saúde em domicílio, afetaram as ações de vacinação, que necessitam o deslocamento ao serviço de saúde^22^. A preocupação dos pais de expor as crianças ao Sars-CoV-2 ao levá-las aos serviços de saúde para a vacinação também contribuiu para o declínio das coberturas vacinais^22^^,^^24^^–^^26^^,^^28^. Estudo de risco-benefício em países africanos mostrou que as mortes evitáveis pela vacinação de rotina superam o excesso de risco de morte por covid-19 associado ao comparecimento no serviço de saúde para a vacinação, evidenciando a necessidade de esforços voltados a aumentar as coberturas vacinais neste momento^21^.

As coberturas vacinais infantis caíram acentuadamente durante a pandemia em diversas regiões do mundo^26^^,^^28^. Nos EUA foi encontrado um declínio considerável das coberturas vacinais de crianças, com início na semana após a declaração de emergência nacional (13 de março de 2020), sendo maior entre os menores de dois anos de idade^25^. Na Inglaterra, três semanas após a introdução do distanciamento social (20 de março de 2020), houve queda de 19,8% das doses aplicadas da vacina de sarampo-caxumba-rubéola, comparando-se com o mesmo período em 2019^24^. Em Michigan (EUA), a completude do esquema vacinal de crianças aos cinco meses de idade caiu de 67,0% para 49,7% em maio de 2020. Aos 16 meses, verificou-se que a cobertura da vacina de sarampo caiu de 76,1% para 70,9%^22^. Na Indonésia, onde a imunização ocorre nas escolas, estimou-se uma queda importante da cobertura do esquema básico vacinal após o fechamento das escolas, em março de 2020^27^. Ademais, sabe-se que esse impacto será ainda mais importante em famílias de condições socioeconômicas desfavoráveis^26^.

A Organização Mundial da Saúde (OMS) estima que pelo menos 80 milhões de crianças estarão suscetíveis a doenças imunopreveníveis como sarampo, difteria e poliomielite por conta da queda das coberturas vacinais durante a pandemia de covid-19^29^. Vale lembrar que surtos de sarampo foram atribuídos à interrupção de serviços de vacinação durante a epidemia de ebola de 2013–2016 na África Ocidental, ocasionando uma segunda crise de saúde pública^30^^–^^31^.

A pandemia no novo coronavírus desafiou os sistemas de saúde do mundo todo na prestação de serviços essenciais, incluindo os programas de imunização, pois a vacinação de rotina e as campanhas de vacinação em massa poderiam contribuir para a disseminação da covid-19^32^.

Em 26 de março de 2020, a OMS e a Organização Pan-Americana da Saúde publicaram recomendações sobre a vacinação durante a pandemia de covid-19. As medidas consideravam três cenários de disponibilidade dos serviços de saúde e incluíam a suspensão temporária das campanhas de vacinação em massa durante esse período. Recomendou-se que a vacinação de rotina fosse mantida em locais onde os serviços essenciais de saúde tivessem capacidade operacional de recursos humanos e fornecimento de vacinas preservados, respeitando-se o distanciamento físico e outras medidas de controle da transmissão do Sars-CoV-2^33^^,^^34^.

No Brasil, houve a recomendação de suspender a imunização de rotina nos primeiros 15 dias após o início da campanha de vacinação de influenza, período destinado aos grupos de idosos e profissionais da saúde, o que, apesar de válido para a proteção de idosos, gerou preocupação por parte de sociedades médicas brasileiras^35^.

A OMS reconhece essa fragilidade e recomenda esforços para garantir altas coberturas vacinais, buscando-se a proteção de rebanho para doenças imunopreveníveis, de forma que os programas de imunização devem adotar medidas inovadoras^36^^,^^37^. Estratégias de vacinação em veículos, em casa ou em salas específicas e bem separadas dos locais dos demais atendimentos clínicos poderiam ser utilizadas, bem como a identificação de faltosos e o recrutamento para a vacinação com o auxílio de registros informatizados de imunização (RII)^22^^,^^24^^–^^26^^,^^28^.

Os RII permitem maior eficiência dos serviços de saúde, pois, além de proporcionarem a avaliação de coberturas vacinais, também auxiliam na prática rotineira e viabilizam a convocação de faltosos, aumentando assim a abrangência da imunização^38^^,^^39^. Além disso, constituem importantes fontes de informação, que podem ser aplicadas na avaliação de indicadores de desempenho e no desenvolvimento de pesquisas epidemiológicas^38^^,^^40^. Em 2020, pesquisadores de países como EUA e Reino Unido avaliaram em tempo real a queda das coberturas vacinais e do número de doses aplicadas durante a pandemia da covid-19 por meio de RII. Com essa ágil identificação é possível adotar rapidamente estratégias ante esse desafio^22^^,^^24^^,^^25^.

Com o surgimento, em muitos países, de movimentos radicais negando a importância conferida à pandemia e contrários às medidas de mitigação do evento, a importância da hesitação vacinal pode ser ampliada, tendo em vista a possível disponibilidade, num futuro próximo, de novas vacinas para a prevenção da covid-19, que terão papel importante no enfrentamento dessa doença^41^.

## DESAFIOS DO RETORNO ÀS ESCOLAS

A retomada segura das creches e escolas deve ser uma prioridade nacional. As crianças perderam benefícios sociais, educacionais e de desenvolvimento elementares, e para muitos pais não será possível o retorno ao trabalho caso essas instituições permaneçam fechadas, exacerbando as iniquidades sociais. Diversas medidas individuais (uso de máscara, higiene, distanciamento social, medição de temperatura, entre outras) e ambientais (capacidade e disposição das salas de aula, limpeza, entre outras) serão necessárias para prevenir a transmissão do Sars-CoV-2 entre escolares e funcionários, inclusive no transporte até as escolas^42^^,^^43^.

Entretanto, além dos cuidados em relação a própria covid-19^44^, a *American Academy of Pediatrics* recomendou que escolas, serviços de saúde e autoridades sanitárias locais promovam a vacinação infantil bem antes do início do ano letivo. Ressalta-se a importância de que as crianças recebam as vacinas na idade preconizada e sejam atualizadas em caso de atraso vacinal devido à pandemia^45^. Tal recomendação deveria ser considerada em outros países, inclusive no Brasil.

No país, a pandemia constituiu um desafio adicional para o retorno às escolas devido às doenças imunopreveníveis, pois, conforme já exposto, há alguns anos enfrentávamos uma queda consistente das coberturas vacinais e uma ampla epidemia de sarampo, atingindo vários estados e totalizando milhares de casos. Esta situação piora em 2020, que até agosto havia registrado mais de 7 mil casos confirmados de sarampo^46^.

Segundo dados do Sistema de Informação do PNI (SI-PNI), ao compararmos o número de primeiras doses aplicadas da vacina pentavalente em março de 2020 com o mesmo mês do ano anterior, verificamos uma queda de 27% (Figura)^10^^–^^11^. Tais dados apontam que o retorno às aulas poderá ampliar o risco não só de expansão das epidemias de sarampo em todo o país, mas também de reemergência de outras já controladas, das quais vale salientar a difteria e a coqueluche. Estudos mostram que surtos de difteria ocorrem quando há queda das coberturas vacinais devido a migrações e/ou instabilidade política, ressaltando ser uma doença de letalidade importante^47^^–^^49^.

**Figura:** Número de doses aplicadas da vacina pentavalente* (primeira dose) no Brasil, segundo mês e ano (2015–2020).

Fonte: SI-PNI (http://sipni.datasus.gov.br).

Assim, fica evidente que, antes do retorno progressivo das atividades escolares presenciais, serão necessárias ações intensivas de avaliação da situação vacinal dessa população com o intuito de recuperar coberturas vacinais suficientes para impedir ou reduzir a disseminação de doenças imunopreveníveis^45^. Instrumentos inovadores, como os RII, podem ser úteis para a avaliação em tempo real das coberturas vacinais, bem como para alertar sobre a imunização e resgatar indivíduos com atraso vacinal^22^^,^^24^^,^^25^^,^^38^^–^^40^.

## CONSIDERAÇÕES FINAIS

No Brasil, até o momento não há estudos sobre o impacto da covid-19 na queda das coberturas vacinais. Também não foi investigado, mesmo em outros países, o atraso vacinal de crianças que deveriam ter sido imunizadas no momento mais intenso do distanciamento social. Além disso, apesar da universalidade do acesso à vacinação infantil alcançada pelo PNI na última década, provavelmente esse impacto será maior em crianças de famílias com condições socioeconômicas desfavoráveis, devido ao menor acesso aos serviços e às informações de saúde.

Quando as medidas de distanciamento social forem afrouxadas, muitas crianças estarão suscetíveis a doenças imunopreveníveis, e haverá a necessidade de avaliar a situação vacinal dos escolares antes da volta às aulas^22^^,^^24^^–^^26^^,^^28^^,^^45^.

A pandemia de covid-19 relembrou a importância da vacinação ao mostrar o quão rápido uma doença pode se espalhar e causar danos irreparáveis na sociedade sem essa defesa. No momento em que uma vacina segura e eficaz para o Sars-CoV-2 estiver disponível, os programas de imunização terão um desafio ainda maior de se fortalecer e alcançar os mais vulneráveis^28^.
